# Development and validation of the VitaL CLASS score to predict mortality in stage IV solid cancer patients with septic shock in the emergency department: a multi-center, prospective cohort study

**DOI:** 10.1186/s12916-020-01875-5

**Published:** 2020-12-14

**Authors:** Youn-Jung Kim, Jihoon Kang, Min-Ju Kim, Seung Mok Ryoo, Gu Hyun Kang, Tae Gun Shin, Yoo Seok Park, Sung-Hyuk Choi, Woon Yong Kwon, Sung Phil Chung, Won Young Kim, on behalf of the Korean Shock Society (KoSS) Investigators

**Affiliations:** 1 Department of Emergency Medicine, University of Ulsan College of Medicine, Asan Medical Center, 88, Olimpic-ro 43-gil, Songpa-gu, Seoul, 05505 South Korea; 2 Department of Hematology/Oncology, Department of Internal Medicine, Kangbuk Samsung Medical Center, Sungkyunkwan University School of Medicine, Seoul, South Korea; 3 Department of Clinical Epidemiology and Biostatistics, Asan Medical Center, Seoul, South Korea; 4 Department of Emergency Medicine, Hallym University College of Medicine, Seoul, South Korea; 5 Department of Emergency Medicine, Samsung Medical Center, Sungkyunkwan University School of Medicine, Seoul, South Korea; 6 Department of Emergency Medicine, Yonsei University College of Medicine, Seoul, South Korea; 7 Department of Emergency Medicine, Guro Hospital, Korea University Medical Center, Seoul, South Korea; 8 Department of Emergency Medicine, Seoul National University College of Medicine, Seoul, South Korea

**Keywords:** Septic shock, Sepsis, Neoplasms, Prognosis, Critical care

## Abstract

**Background:**

Clinical decision-making of invasive high-intensity care for critically ill stage IV cancer patients in the emergency department (ED) is challenging. A reliable and clinically available prognostic score for advanced cancer patients with septic shock presented at ED is essential to improve the quality of intensive care unit care. This study aimed to develop a new prognostic score for advanced solid cancer patients with septic shock available early in the ED and to compare the performance to the previous severity scores.

**Methods:**

This multi-center, prospective cohort study included consecutive adult septic shock patients with stage IV solid cancer. A new scoring system for 28-day mortality was developed and validated using the data of development (January 2016 to December 2017; *n* = 469) and validation sets (January 2018 to June 2019; *n* = 428). The developed score’s performance was compared to that of the previous severity scores.

**Results:**

New scoring system for 28-day mortality was based on six variables (score range, 0–8): vital signs at ED presentation (respiratory rate, body temperature, and altered mentation), lung cancer type, and two laboratory values (lactate and albumin) in septic shock (VitaL CLASS). The C-statistic of the VitaL CLASS score was 0.808 in the development set and 0.736 in the validation set, that is superior to that of the Sequential Organ Failure Assessment score (0.656, *p* = 0.01) and similar to that of the Acute Physiology and Chronic Health Evaluation II score (0.682, *p* = 0.08). This score could identify 41% of patients with a low-risk group (observed 28-day mortality, 10.3%) and 7% of patients with a high-risk group (observed 28-day mortality, 73.3%).

**Conclusions:**

The VitaL CLASS score could be used for both risk stratification and as part of a shared clinical decision-making strategy for stage IV solid cancer patients with septic shock admitting at ED within several hours.

**Supplementary Information:**

The online version contains supplementary material available at 10.1186/s12916-020-01875-5.

## Background

Cancer is a major public health burden; both cancer incidence and mortality are expected to increase rapidly worldwide. Recent advances in cancer treatment have improved the overall survival rates; however, they have also increased the possibility of developing a critical illness requiring intensive care unit (ICU) management [1, 2]. Approximately 5% of the patients with solid cancer require ICU admission within 2 years after diagnosis [1, 2].

Sepsis and septic shock associated with cancer progression or chemo-radiation-therapy is a common life-threatening complication in cancer patients. Recent studies have demonstrated improved outcomes in cancer patients admitted to the ICU, and with appropriate patient selection, the outcomes of patients with or without cancer could be similar [3–5]. However, the decision of invasive ICU treatments for advanced cancer patients with septic shock is still challenging, especially in the emergency department (ED) setting. The clinical decision-making for advanced cancer patients with septic shock in the ED comprises more than 3 of health care specialties: critical care medicine, emergency medicine, oncology, and surgery. Treatment recommendations for these patients could be different depending on the specialties [6, 7]. Also, the patient-physician communication in such situations frequently leads to overutilization of invasive ICU treatments, which can produce more costly and invasive care without improving outcomes [8–10].

Sequential Organ Failure Assessment (SOFA) and Acute Physiology and Chronic Health Evaluation (APACHE) II scores are valid and widely used clinical prediction tools to determine the mortality risk, but they are designed to be calculated on the worst parameters recorded during the initial 24 h after admission and are not reliable at ED presentation [11, 12]. A reliable and clinically available prognostic score for advanced cancer patients with septic shock presented at ED is essential to improve the quality and efficiency of ICU care. To address this issue, we aimed to develop a new prognostic model for stage IV cancer patients who present with septic shock available at ED and compare its performance to existing scoring systems including SOFA score, APACHE II score, quick SOFA score, National Early Warning Score, and Modified Early Warning Score.

## Methods

### Study design and population

This multi-center, prospective observational study was conducted in the EDs of 11 Korean university-affiliated, tertiary referral centers using data from the Korean Shock Society septic shock registry, from January 2016 to December 2018. The Korean Shock Society is a Korean collaborative research network, established in 2013, for improving the quality of research, diagnosis, and management of sepsis [13]. Since October 2015, 11 EDs in the Korean Shock Society have been prospectively collecting data pertaining to patients with septic shock [13]. Adult patients (aged ≥ 19 years) who visited one of these EDs with suspected or confirmed infection and evidence of refractory hypotension or hypoperfusion were enrolled in the registry [14–16]. Refractory hypotension was defined as persistent systolic blood pressure < 90 mmHg, mean arterial pressure < 70 mmHg, or systolic blood pressure decrease > 40 mmHg after ≥ 20–30 mL/kg intravenous fluid administration or requiring vasopressors to maintain systolic blood pressure of ≥ 90 mmHg or mean arterial pressure of ≥ 70 mmHg [17]. Hypoperfusion was defined as serum lactate level of ≥ 4 mmol/L [18]. Exclusion criteria included patients who refused ICU management, patients who signed a “do not attempt resuscitation” order before ED arrival or at the time of diagnosis, patients who met the inclusion criteria 6 h after ED arrival, patients who were transferred from other hospitals after stabilization, patients who were directly transferred to other hospitals at ED, or patients who refused to enroll in the registry [13]. The institutional review boards of each participating institute approved the registry, and informed consent was obtained before data collection.

This study included patients with stage IV solid cancer who were enrolled in the septic shock registry and treated with intensive care between 1 January 2016 and 30 June 2019. The final cohort for this study included 897 patients; those selected from 1 January 2016 to 31 December 2017 were included in the development set (*n* = 469, 52.3%) and those selected from 1 January 2018 to 30 June 2019, in the validation set (*n* = 428, 47.7%).

### Management and data collection

All patients were treated in accordance with then-current guidelines of the Surviving Sepsis Campaign including crystalloid administration, acquisition of blood cultures before antibiotic administration, and broad-spectrum antibiotic administration and vasopressor administration [17, 19]. The decision for subsequent intensive care was based on the intensivist according to institutional protocols. Empirical broad-spectrum antibiotics, such as piperacillin/tazobactam, cefepime, and meropenem, were administrated immediately after blood cultures at the ED, and after admission, further antibiotic treatments were adjusted.

The case report form of the Korean Shock Society septic shock registry includes standard definitions of 200 variables including demographic and clinical characteristics, therapeutic interventions, and outcomes. Data of the Korean Shock Society septic shock registry are collected via a standardized case report form and entered into a web-based electronic database [13]. The quality management committee monitors and reviews the completeness and consistency of data regularly and also gives feedback to the research coordinators and investigators of the results of the quality management process by using the web system’s query function or via a telephone call.

Data regarding age, sex, comorbid disease, focus of infection, laboratory findings, vital signs at ED presentation, and SOFA and APACHE II scores were retrieved from the registry. Mental status at ED presentation was assessed using the Alert/responsive to Voice/responsive to Pain/Unresponsive scale; unalert patients were considered to have altered mentation. Cancer type was reviewed additionally for this study using the diagnosis codes according to the International Statistical Classification of Diseases and Related Health Problems, Tenth Revision. We categorized the cases according to six common solid cancer types as follows: gastrointestinal cancer (C15-C20), hepatobiliary-pancreas cancer (C22-C25), lung cancer (C33-C34), gynecologic cancer (C53-C56), urologic cancer (C61-67), and “others/ill-defined” cancer. All blood samples for laboratory analysis including lactic acid were obtained from the patients at their initial presentation. SOFA and APACHE II scores were calculated using the worst parameters during the initial 24 h after ED admission. The quick SOFA score, National Early Warning Score, and Modified Early Warning Score were calculated using the initial vital signs at ED admission [20–22]. The primary endpoint of this study was 28-day mortality.

### Statistical analysis

Categorical variables were presented as frequencies and percentages, and continuous variables as median with interquartile range (IQR). The *χ*^2^ test was used to compare categorical variables of development and validation sets, and the continuous variables for survivors and non-survivors of development and validation sets were compared using the *t* test or the Wilcoxon rank-sum test if the distribution was not normal. Lactic acid level was categorized into 3 groups (twice above the normal limit [≥ 4 mmol/L], 4 to 8 mmol/L, fourfold or more above the normal limit [≥ 8 mmol/L]) based on the previous guidelines and study [17, 23].

In the development set, univariate Cox proportional hazard analysis was performed to evaluate the prognostic ability of each variable. The variables in the scoring system were selected among all variables by using multivariable Cox proportional hazard analysis with backward elimination in 1000-fold bootstrap resampling [24]. Then, we counted how many times each candidate variable remained in the model in the 1000 bootstrap samples. If a variable appeared > 500 times in the final model, the variable was included in the scoring system. To allocate points in the scoring system, the bootstrapping method was used again to obtain bias-corrected regression coefficients to assign risk points in the scoring system. Risk points were obtained by bias-corrected regression coefficients multiplied by a reference value in the corresponding category. Each risk point was rescaled to designate the point of lung cancer as 1 (for example, the risk point for respiratory rate ≥ 22/min was 1, which was rounded from 0.891/0.617 = 1.444). A reference risk factor profile was chosen by selecting a base category for each risk factor, which was assigned 0 points in the scoring system. The total score was the weighted sum of those predictors of which the weights were defined as the rounded integer value of the quotient of regression coefficients divided by the regression coefficient of the reference predictor. The total scores based on patient profiles were converted into a risk estimate by using a specific formulation as below [25].

$$ \hat{p}=1-{S}_0{(t)}^{\exp \left({\sum}_{i=1}^p{\beta}_i{X}_i-{\sum}_{i=1}^p{\beta}_i{\overline{X}}_i\right)} $$

The calibration and discrimination of the final multivariable Cox proportional hazards model were assessed by calibration curve [26] and the Harrell C-statistic [27], respectively. The calibration measure compared the predicted and observed probability after data were partitioned into several groups. The discrimination index measured the concordance probability that predicted probabilities from a randomly selected pair of survivors and non-survivors. We categorized the groups based on the likelihood of 28-day mortality: low (score of 0–2, 7.07%), average (score of 3–5, 36.03%), and high (score of 6–8, 89.47%). Finally, we compared the C-index and Akaike information criterion of our new prognostic score with those of the SOFA and APACHE II scores using R package “compareC” [28].

All reported *p* values were 2-sided, and *p* < 0.05 was considered significant. All statistical analyses were performed using the SAS version 9.4 (SAS Institute Inc., Cary, NC, USA) and R (version 3.6.1; R Foundation for Statistical Computing, Vienna, Austria; https://www.R-project.org).

## Results

During the study period, a total of 3486 patients with septic shock were enrolled in the Korean Shock Society septic shock registry, of which 897 (25.7%) patients with stage IV solid cancer were included. The overall 28-day mortality rate was 26.4% (237/897) and 499 (55.6%) patients admitted to ICU. The general characteristics of our study patients including those in the development (*n* = 469, 52.3%) and validation (*n* = 428, 47.7%) cohorts are presented in Table 1. The median age of our cohort was 66 years, and hepatobiliary-pancreas cancer (31.9%) and lung cancer (16.8%) were the dominant cancer types. Patients in the validation set showed significantly higher initial lactate level (median, 3.4 vs. 3.7 mmol/L, *p* = 0.03), SOFA (median, 7.0 vs. 8.0, *p* = 0.002), and APACHE II (median, 19.0 vs. 21.0, *p* = 0.04) scores.

**Table 1:** Characteristics of the patients in the development set and validation set

Characteristics	Total set (*N* = 897)	Development set (*n* = 469)	Validation set (*n* = 428)	*p* value
Age, years	66.0 (59.0–74.0)	67.0 (60.0–74.0)	66.0 (59.0–75.0)	0.38
Male	561 (62.5)	292 (62.3)	269 (62.9)	0.86
Hypertension	281 (31.3)	143 (30.5)	138 (32.2)	0.57
Diabetes mellitus	207 (23.1)	122 (26.0)	85 (19.9)	0.09
Cancer type				0.46
Gastrointestinal	137 (15.3)	62 (13.2)	75 (17.5)	
Hepatobiliary-pancreas	286 (31.9)	151 (32.2)	135 (31.5)	
Lung	151 (16.8)	79 (16.8)	72 (16.8)	
Gynecologic	82 (9.1)	49 (10.5)	33 (7.7)	
Urologic	78 (8.7)	42 (9.0)	36 (8.4)	
Others	163 (18.2)	86 (18.3)	77 (18.0)	
Focus of infection				0.21
Pneumonia	209 (23.3)	106 (22.6)	103 (24.1)	
Urinary tract infection	116 (12.9)	65 (13.9)	51 (11.9)	
Colitis	129 (14.4)	59 (12.6)	70 (16.4)	
Cholangitis/cholecystitis	238 (26.5)	130 (27.7)	108 (25.2)	
Others/unknown	129 (14.4)	75 (16.0)	54 (12.6)	
Multiple focus	76 (8.5)	34 (7.3)	42 (9.8)	
Vital signs at ED admission				
Systolic BP, mmHg	90.0 (77.0–111.0)	90.0 (79.0–112.0)	89.0 (76.0–110.0)	0.23
Diastolic BP, mmHg	56.0 (48.0–66.0)	57.0 (48.0–68.0)	56.0 (48.0–64.5)	0.17
Heart rate/min	116.0 (97.0–132.0)	116.0 (97.0–131.0)	115.0 (98.0–134.0)	0.50
Respiratory rate/min	20.0 (18.0–22.0)	20.0 (18.0–23.0)	20.0 (18.0–22.0)	0.25
Body temperature, °C	37.7 (36.8–38.7)	37.8 (36.9–38.8)	37.6 (36.7–38.5)	0.005
Altered mentation	115 (12.8)	71 (15.1)	44 (10.3)	0.03
Laboratory values				
White blood cell, /μL	8100 (3000–15,360)	8000 (2870–15,300)	8300 (3140–15,545)	0.56
Hemoglobin, g/dL	10.2 (8.6–11.6)	10.2 (8.5–11.6)	10.1 (8.8–11.8)	0.19
Platelet count, × 1000/μL	143.0 (71.0–232.0)	143.0 (68.0–228.0)	142.5 (77.0–242.5)	0.53
PT, INR	1.29 (1.15–1.47)	1.28 (1.17–1.46)	1.30 (1.15–1.48)	0.96
Albumin, g/dL	2.8 (2.3–3.2)	2.8 (2.4–3.3)	2.7 (2.3–3.2)	0.21
BUN, mg/dL	24.6 (17.4–38.3)	24.3 (17.0–36.0)	25.0 (18.0–41.0)	0.17
Creatinine, mg/dL	1.25 (0.89–1.90)	1.19 (0.87–1.85)	1.32 (0.91–1.95)	0.06
CRP, mg/dL	12.8 (6.1–22.1)	12.3 (6.5–21.7)	13.2 (5.6–23.6)	0.55
Lactic acid, mmol/L	3.6 (2.0–5.5)	3.4 (1.9–5.3)	3.7 (2.2–5.8)	0.03
Severity score				
SOFA score	8.0 (6.0–10.0)	7.0 (5.0–10.0)	8.0 (6.0–11.0)	0.002
APACHE II score	20.0 (15.0–26.0)	19.0 (14.0–25.0)	21.0 (15.0–27.0)	0.04
Quick SOFA score	1.0 (1.0–2.0)	1.0 (1.0–2.0)	1.0 (1.0–2.0)	0.50
NEWS	7.0 (5.0–10.0)	7.0 (5.0–10.0)	7.0 (5.0–9.0)	0.99
MEWS	5.0 (4.0–7.0)	5.0 (4.0–7.0)	5.0 (4.0–6.0)	0.75
ICU admission	499 (55.6)	246 (52.5)	253 (59.1)	0.05
28-day mortality	237 (26.4)	120 (25.6)	117 (27.3)	0.55

Values are presented as median (interquartile range) or number (percentage), as appropriate

*Abbreviations*: *APACHE* Acute Physiology and Chronic Health Evaluation, *BP* blood pressure, *BUN* blood urea nitrogen, *CRP* C-reactive protein, *ED* emergency department, *ICU* intensive care unit, *INR* international normalized ratio, *MEWS* Modified Early Warning Score, *NEWS* National Early Warning Score, *PT* prothrombin time, *SOFA* Sequential Organ Failure Assessment

**Fig. 1:**
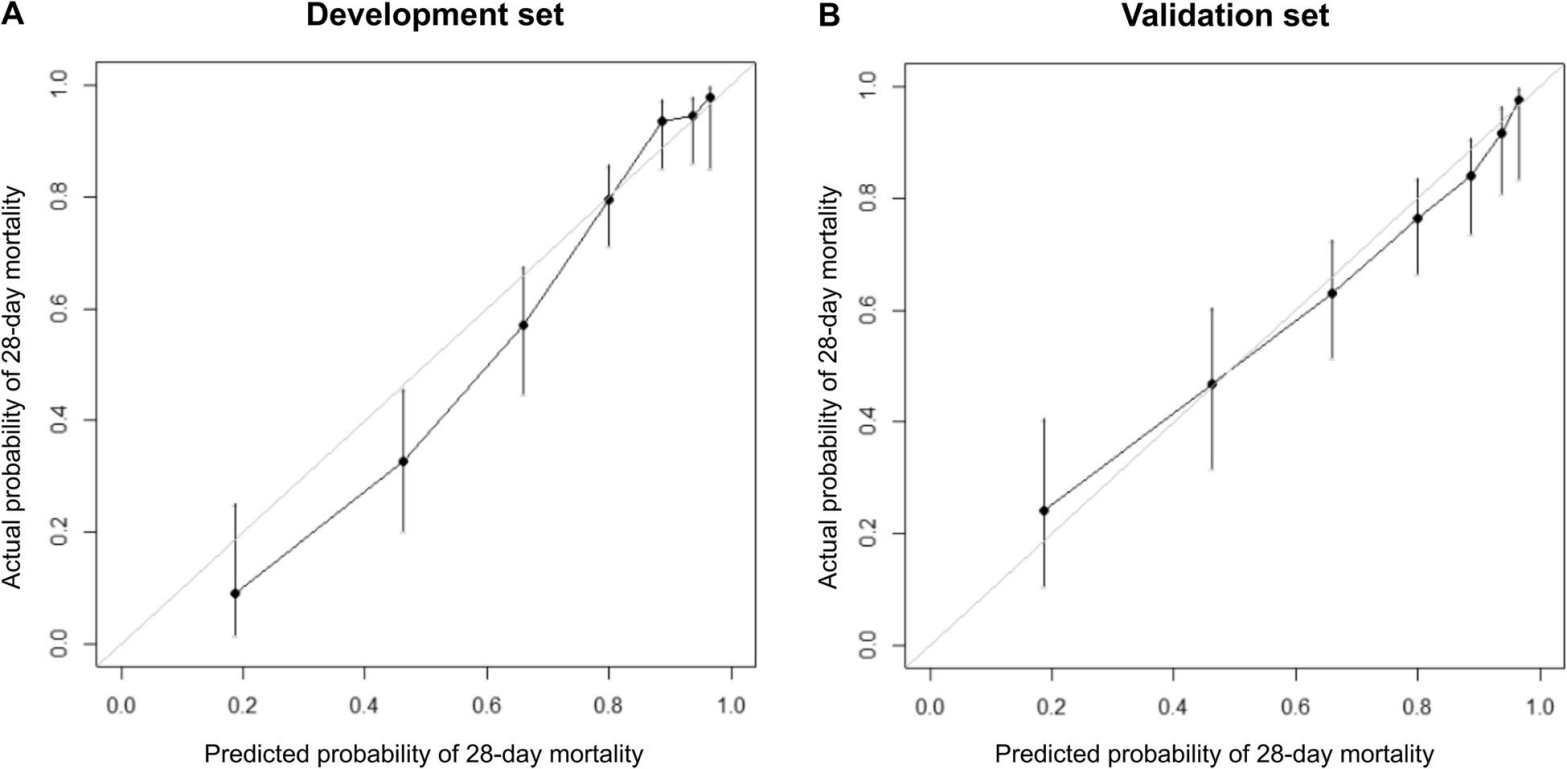
Internal validation in the development set (**a**) and external validation in the validation set (**b**)

The univariable Cox proportional hazards model for predicting the 28-day mortality in the development set is summarized in Table 2. The bootstrap resampling variable selection method provided six variables: respiratory rate, body temperature, altered mentation at ED presentation, lung cancer, lactic acid, and albumin. We performed a second multivariate Cox proportional hazards analysis using only those six final independent variables, and the final prediction model was developed based on the bias-corrected regression coefficients as shown in Tables 3 and 4. We named this new prognostic scoring system the VitaL CLASS score (vital signs-lung cancer-lactate-albumin in septic shock). The C-statistic for the VitaL CLASS score was 0.808 (95% confidence interval [CI], 0.772–0.845) in the development set and 0.736 (95% CI, 0.694–0.778) in the validation set (Table 5). In the development set, the C-static for the VitaL CLASS score was superior to that of the existing scoring systems such as the SOFA (0.713; 95% CI, 0.664–0.762; *p* = 0.001) and APACHE II (0.692; 95% CI, 0.643–0.741; *p* < 0.001). In the validation set, the C-statistic of the VitaL CLASS score (0.736) was superior to that of the SOFA score (0.656; *p* = 0.01) and similar to that of the APACHE II score (0.682; *p* = 0.08). The calibration curve of our new score demonstrated good correlation between the predicted and actual probability of 28-day mortality in both sets (Fig. 1). A subgroup analysis of the patients who fulfill the sepsis-3 criteria for septic shock was performed. Additional file 1: Table S1 shows there was no difference in general characteristics between development and validation subgroup cohorts. The VitaL CLASS score of the patients who fulfill the sepsis-3 criteria for septic shock demonstrated superior prognostic performance to the existing scoring systems in the development set (C-index, 0.806; 95% CI, 0.761–0.851), and similar performance to SOFA and APACHE II scores in the validation set (C-index, 0.702; 95% CI, 0.649–0.755) (Additional file 2: Table S2).

**Table 2:** Characteristics of the survivors and non-survivors and the univariable Cox proportional hazards model for 28-day mortality in the development set

Characteristics	Comparison		Univariable analysis		
	Survivors (*n* = 349)	Non-survivors (*n* = 120)	HR	95% CI	*p* value
Age, years	66.0 (60.0–74.0)	69.0 (60.0–75.0)	1.018	1.000–1.036	0.05
Sex					
Male	210 (60.2)	82 (68.3)	1.000		
Female	139 (39.8)	38 (31.7)	0.739	0.503–1.086	0.12
Hypertension					
No	250 (71.6)	76 (63.3)	1.000		
Yes	99 (28.4)	44 (36.7)	1.382	0.954–2.004	0.09
Diabetes mellitus					
No	260 (74.5)	87 (72.5)	1.000		
Yes	89 (25.4)	33 (27.7)	1.144	0.766–1.707	0.51
Cancer type					
Lung	40 (11.5)	39 (32.5)	1.000		
Hepatobiliary-pancreas	122 (35.0)	29 (24.2)	0.329	0.203–0.533	< 0.001
Gastrointestinal	50 (14.3)	12 (10.0)	0.325	0.170–0.622	0.001
Gynecologic	44 (12.6)	5 (4.2)	0.164	0.065–0.416	< 0.001
Urologic	33 (9.5)	9 (7.5)	0.362	0.175–0.748	0.006
Others	60 (17.2)	26 (21.7)	0.548	0.334–0.901	0.02
Focus of infection					
Pneumonia	61 (17.5)	45 (37.5)	1.000		
Urinary tract infection	53 (15.2)	12 (10.0)	0.390	0.206–0.738	0.004
Colitis	45 (12.9)	14 (11.7)	0.519	0.285–0.945	0.03
Cholangitis/cholecystitis	106 (30.4)	24 (20.0)	0.387	0.235–0.635	< 0.001
Others/unknown	63 (18.1)	12 (10.0)	0.329	0.174–0.622	0.001
Multiple focus	21 (6.0)	13 (10.8)	0.878	0.474–1.627	0.68
Vital signs at ED admission					
Systolic BP, mmHg	90.0 (79.0–110.0)	90.0 (78.0–118.5)	1.004	0.998–1.011	0.20
Diastolic BP, mmHg	57.0 (48.0–66.0)	56.0 (49.0–70.0)	1.005	0.995–1.014	0.33
Heart rate/min	115 (97–131)	117 (96–132)	1.001	0.993–1.008	0.84
Respiratory rate/min	20 (18–22)	22 (20–26)	1.084	1.057–1.113	< 0.001
Body temperature, °C	38.1 (37.1–39.0)	37.3 (36.6–37.9)	0.638	0.546–0.745	< 0.001
Altered mentation	36 (50.7)	35 (49.3)	2.988	2.014–4.431	< 0.001
Laboratory values					
White blood cell, /μL	7890 (2740–14,300)	9815 (4365–17,570)	1.000	1.000–1.000	0.04
Hemoglobin, g/dL	10.2 (8.5–11.6)	9.8 (8.4–11.5)	0.994	0.915–1.080	0.88
Platelet count, × 1000/μL	144 (70–228)	140 (66–224)	1.000	0.998–1.001	0.69
PT, INR	1.25 (1.14–1.43)	1.39 (1.25–1.61)	1.229	1.075–1.405	0.003
Albumin, g/dL	2.9 (2.5–3.3)	2.5 (2.2–3.0)	0.451	0.334–0.610	< 0.001
BUN, mg/dL	23 (16–33)	30 (19–44)	1.012	1.005–1.018	0.001
Creatinine, mg/dL	1.16 (0.87–1.80)	1.29 (0.84–2.19)	1.104	0.987–1.233	0.08
CRP, mg/dL	12.0 (5.8–20.6)	14.0 (7.6–25.4)	1.011	0.997–1.026	0.13
Lactic acid, mmol/L	3.1 (1.8–4.9)	4.6 (2.5–8.3)	1.177	1.127–1.228	< 0.001
Severity score^a^					
SOFA score	7.0 (5.0–9.0)	10.0 (7.0–13.0)	1.246	1.190–1.305	< 0.001
APACHE II score	18.0 (14.0–23.0)	24.0 (18.0–32.0)	1.088	1.069–1.108	< 0.001

Values are presented as median (interquartile range) or number (percentage), as appropriate

*Abbreviations*: *APACHE* Acute Physiology and Chronic Health Evaluation, *BP* blood pressure, *BUN* blood urea nitrogen, *CI* confidence interval, *CRP* C-reactive protein, *ED* emergency department, *HR* hazard ratio, *INR* international normalized ratio, *PT* prothrombin time, *SOFA* Sequential Organ Failure Assessment

^a^Severity scores were not included in the multivariate analysis for the final model

**Table 3:** Multivariable analysis model for 28-day mortality in the development set

Characteristics	Multivariable analysis			Bootstrapping method
	HR	95% confidence interval	*p* value	Relative frequency*
Lung cancer	2.025	1.345–3.050	0.001	0.959
Vital signs at ED admission				
Respiratory rate ≥ 22/min	2.517	1.713–3.698	< 0.001	0.858
Body temperature < 38.0 °C	2.918	1.903–4.475	< 0.001	0.994
Altered mentation	2.021	1.328–3.074	0.001	0.923
Laboratory values				
Albumin < 2.8 g/dL	2.473	1.665–3.672	< 0.001	0.977
Lactic acid, mmol/L			< 0.001	
4.0–7.9	1.597	1.032–2.473	0.04	0.997
≥ 8.0	3.767	2.343–6.054	< 0.001	

*HR* hazard ratio

*A 50% relative frequency of selection in bootstrap resampling was the criterion for inclusion of predictors in the final multivariate model

**Table 4:** The new prognostic scoring system named VitaL CLASS score (vital signs-lung cancer-lactate-albumin in septic shock) predicting for 28-day mortality

Risk factor	Category	Bias-corrected regression coefficient	Points*
Lung cancer	No	0	0
	Yes	0.617	1
Vital signs at ED admission			
Respiratory rate	< 22/min	0	0
	≥ 22/min	0.891	1
Body temperature	≥ 38.0 °C	0	0
	< 38.0 °C	1.016	2
Mentation	Alert	0	0
	Alteration	0.645	1
Laboratory values			
Albumin	≥ 2.8 g/dL	0	0
	< 2.8 g/dL	0.864	1
Lactic acid, mmol/L	< 4.0	0	0
	4.0–7.9	0.446	1
	≥ 8.0	1.228	2

*ED* emergency department

Total score ranges 0–8

*Risk points were obtained by bias-corrected regression coefficients and reference values in each category. Each risk point was rescaled to designate the point of lung cancer as 1

**Table 5:** The C-indices for testing of the VitaL CLASS score and other pre-existing scoring systems

Scoring system	Development set			Validation set		
	AIC	C-index (95% CI)	*p* value	AIC	C-index (95% CI)	*p* value
VitaL CLASS score	1287.4	0.808 (0.772–0.845)	Reference	1299.1	0.736 (0.694–0.778)	Reference
SOFA score	1362.0	0.713 (0.664–0.762)	0.001	1347.2	0.656 (0.603–0.708)	0.01
APACHE II score	1370.3	0.692 (0.643–0.741)	< 0.001	1329.1	0.682 (0.633–0.731)	0.08
Quick SOFA score	1419.2	0.614 (0.565–0.663)	< 0.001	1380.4	0.552 (0.502–0.601)	< 0.001
NEWS	1399.2	0.669 (0.617–0.720)	< 0.001	1379.3	0.561 (0.509–0.613)	< 0.001
MEWS	1435.5	0.554 (0.501–0.607)	< 0.001	1383.6	0.486 (0.433–0.540)	< 0.001

*AIC* Akaike information criterion, *APACHE* Acute Physiology and Chronic Health Evaluation, *CI* confidence interval, *MEWS* Modified Early Warning Score, *NEWS* National Early Warning Score, *SOFA* Sequential Organ Failure Assessment, *VitaL CLASS* vital signs-lung cancer-lactate-albumin in septic shock

Table 6 shows the performance of the final point score and risk groups according to the VitaL CLASS score in development and validation datasets. Based on the estimate of probability for 28-day mortality, the VitaL CLASS score classified the patients into 3 groups: low (0–2), average (3–5), and high (6–8) risk groups. In the validation set, the VitaL CLASS score identified 40.9% and 7.0% of the patients as having a low risk and high risk of 28-day mortality, respectively. The patients in the low-risk group had 10.3% (18/175) of mortality, whereas 73.3% (22/30) of the patients in the high-risk group died within 28 days after admission.

**Table 6:** Performance of the prognostic model in the development, validation, and combination sets

Total risk points	Estimate of probability	Development set (*n* = 469)		Validation set (*n* = 428)	
		Patients, %	Observed death within 28 days/patients (%)	Patients, %	Observed death within 28 days/patients (%)
0	3.47%	9.6	1/45 (2.2)	9.3	1/40 (2.5)
1	6.33%	15.8	4/74 (5.4)	13.8	5/59 (8.5)
2	11.42%	16.8	5/79 (6.3)	17.8	12/76 (15.8)
3	20.14%	26.7	25/125 (20.0)	22.7	23/97 (23.7)
4	34.10%	15.6	31/73 (42.5)	18.9	30/81 (37.0)
5	53.84%	10.9	34/51 (66.7)	10.5	24/45 (53.3)
6	76.15%	3.4	14/16 (87.5)	5.6	18/24 (75.0)
7	92.99%	0.9	4/4 (100)	1.4	4/6 (66.7)
8	99.28%	0.4	2/2 (100)	0	–
Risk group	Estimate of probability	Patients, %	Observed death within 28 days/patients (%)	Patients, %	Observed death within 28 days/patients (%)
Low (0–2)	7.07%	42.2	10/198 (5.1)	40.9	18/175 (10.3)
Average (3–5)	36.03%	53.1	90/249 (36.1)	52.1	77/223 (34.5)
High (6–8)	89.47%	4.7	20/22 (90.9)	7.0	22/30 (73.3)

## Discussion

We developed and validated a simple and objective prognostic model, the VitaL CLASS score, which is applicable in the ED very early. This scoring system could identify stage IV cancer patients who present to the ED with septic shock as having low, average, or high risk of death within 28 days. Using this score, it would be helpful to triage patients in the high-risk group, who have a low possibility of benefitting from intensive care, and those in the low-risk group, who have a high likelihood of survival. This risk assessment would help physicians share information with patients and family and establish goals-of-care among physicians of different specialties as well as patients in the ED regarding life-sustaining treatments including mechanical ventilation, vasoactive infusions, new renal replacement therapy, or cardiopulmonary resuscitation and physician orders for life-sustaining treatment (POLST) orders. The VitaL CLASS score shows good prognostic performance and is simple and fast to use, comparing to existing scoring systems, SOFA and APACHE II scores.

The VitaL CLASS score to predict short-term mortality among stage IV cancer patients with septic shock consists of four clinical factors identified at ED presentation: cancer type (lung cancer and the other types of cancer), respiratory rate, body temperature and altered mentation, and two laboratory values reported within hours, i.e., lactic acid and albumin. This simple and objective prognostic model for stage IV cancer patients with septic shock uses data available within hours after ED admission. The majority of patients with advanced medical illness would prefer palliative approaches to invasive ICU treatments when they were informed of their therapeutic options, and such invasive ICU treatments should be aligned with patients’ values and prognostic information [10, 29–31]. Furthermore, the demand for ICU care usually exceeds supply, and consequently, the triage and allocation decisions for ICU care for critically ill patients are important. Although objective decision-making guidelines would facilitate the fair use of medical resources, these guidelines are imprecise and are not sufficiently validated for advanced cancer patients [32–34]. Recently, a new strategy for critically ill cancer patients has been introduced considering these limitations that consist of unlimited ICU management with a full-code status for a limited period [35, 36]. However, this is difficult to achieve in several hospitals with a chronic shortage of ICU resources. We believe that the VitaL CLASS score could help physicians identify those who are more likely to benefit from invasive ICU management from those with minimal anticipated benefit, to make better allocations of medical resources, and to discuss do-not-attempt-resuscitation or POLST orders in the ED before admission.

Cancer type is an important prognostic factor for cancer patients, and the mortality rate varies by cancer type in cancer patients with severe sepsis/septic shock [37]. Lung cancer was the second most common cancer type in our cohort (16.8%), following hepatobiliary-pancreas cancer (31.9%). In our study, lung cancer patients with septic shock had the highest 28-day mortality (48.1%) from septic shock, whereas the mortality rate of gynecologic cancer patients was the lowest (10.2%). These mortality rate differences are consistent with those reported in previous studies [37]. In our study, lung cancer was associated with 2.0-fold higher odds when compared with the other types of cancer. Previous studies demonstrated that infection focus, such as respiratory infection, was an important prognostic feature in septic shock patients, but it was not a significant prognostic factor in our study for stage IV solid cancer patients with septic shock [38, 39].

The VitaL CLASS score includes three vital signs at ED admission: respiratory rate, body temperature, and mentation. Tachypnea, i.e., respiratory rate ≥ 22, and altered mentation are commonly used as core poor prognostic factors, consistent with many previous studies on prognostic scoring systems such as quick SOFA, National Early Warning Score, and Modified Early Warning Score [20–22]. Body temperature < 38.0 °C (100.4 °F) was another poor prognostic factor in our prognostic model. Generally, fever is a poor prognostic sign for septic shock patients. However, for cancer patients with septic shock who have a suppressed immune system, fever implies a protective response, namely that their innate and adaptive immune system is functioning better than in those without fever, and such a paradoxical response has been reported in previous studies [40, 41].

The other two laboratory values were lactic acid and albumin levels. Serum lactate levels of ≥ 8.0 mmol/L and from 4.0–7.9 mmol/L were assigned 2 and 1 points, respectively. Lactate level is a reliable biomarker for outcome prediction and severity assessment in numerous conditions [42]. Septic shock according to the Third International Consensus was defined as refractory hypotension and serum lactate level of > 2 mmol/L [43]. A lactate level of > 4 mmol/L at ED admission is associated with mortality in patients with sepsis independent of organ failure, and we used higher cut-offs of 4.0 and 8.0 mmol/L at ED admission [18, 44]. Serum albumin is a surrogate marker of visceral protein function and nutritional status [45], but its synthesis is also suppressed by inflammation [46, 47]. Serum albumin level reflects both the acute and chronic health status of critically ill patients [45, 48]. The cut-off level of albumin (< 2.8 g/dL) in our prediction model is consistent with that used in previous studies, which showed that hypoalbuminemia, defined as < 2.7 or < 2.9 g/dL, was an independent risk factor for mortality in patients with sepsis [48, 49].

The strength of this study was that the VitaL CLASS score consisted of variables that are usually assessed within hours of ED presentation and can be calculated in the time period before admission. However, several limitations should be considered when interpreting the findings of the present study. First, we developed and validated a new prognostic model for stage IV solid cancer patients who present with septic shock to the ED using data from a prospectively collected registry; however, there was no detailed information on cancer-related characteristics such as cancer treatment (i.e., radiotherapy, chemotherapy, and surgery), response to therapy, performance status, and time from initial cancer diagnosis, which are known as major determinant for the short-term outcome. This was a significant limitation of our study. However, recent studies demonstrated that cancer-related characteristics were not associated with short-term mortality [2, 34, 50]. Additionally, performance status is still a valid prognostic factor, but its accurate determination at the ED is challenging due to subjectivity and irreproducibility [51, 52]. Second, the lack of an external validation sample is another main limitation of our study, and further studies for validation of our prognostic model, especially studies involving other races/ethnicities, are warranted. Third, we divided the development (January 2016 to December 2017) and validation sets (January 2018 to June 2019) according to time, and thus, the characteristics of the cohorts and treatments could vary according to the study period. Also, advances in cancer treatment would affect the outcome, which might be associated with the decrease of the predictive value in the validation cohort. However, most variables including outcomes did not show a significant difference, and the study period was only 3.5 years. Additionally, the statistically different variables between development and validation cohorts were not likely to have a clinically significant impact, e.g., body temperature (median, 37.8 vs. 37.6 °C, *p* = 0.005) and lactate level (median, 3.4 vs. 3.7 mmol/L, *p* = 0.03). Fourth, the lactate level could be affected by many clinical conditions such as liver cirrhosis, advanced heart failure, and metformin use. Fifth, all physicians treated patients according to the current Surviving Sepsis guidelines; however, in-hospital treatment strategies, such as ventilator application or continuous renal replacement therapy, and end-of-life decisions that could affect the outcome, were not standardized between the participating hospitals. Lastly, this study did not include the patients who signed a “do not attempt resuscitation” order before ED arrival or at the time of diagnosis, which may lead to a selection bias and limit the generalizability.

## Conclusions

In summary, we developed and validated a simple and objective clinical prediction model, the VitaL CLASS score, for predicting the 28-day mortality in stage IV cancer patients who present with septic shock at the ED. The VitaL CLASS score identified 40.9% of the patients as having a very low likelihood of 28-day mortality (28-day mortality in 10.3%, 18/175) with appropriate critical care and 7.0% of the patients as a high-risk group with minimal anticipated benefit of invasive ICU treatment (28-day mortality in 73.3%, 22 of 30). We believe this prognostic model could provide useful information for patients, their families, and physicians of different specialties and can be used as part of a clinical decision-making strategy regarding aggressive life-sustaining treatments or POLST orders as well as the triage decision for ICU admission.

## Supplementary Information

**Additional file 1: Table S1.** Characteristics of the patients who fulfil the sepsis-3 criteria for septic shock in the development set and validation sets.

**Additional file 2: Table S2.** The C-indices for testing of the VitaL CLASS score and other pre-existing scoring systems in the patients who fulfil the sepsis-3 criteria for septic shock.

## Data Availability

The datasets generated during and/or analyzed during the current study are available from the corresponding author on reasonable request.

## Abbreviations

APACHE: Acute Physiology and Chronic Health Evaluation
CI: Confidence interval
ED: Emergency department
ICU: Intensive care unit
IQR: Interquartile range
POLST: Physician orders for life-sustaining treatment
SOFA: Sequential Organ Failure Assessment
