# Supplementary material for: Development and validation of the VitaL CLASS score to predict mortality in stage IV solid cancer patients with septic shock in the emergency department: a multi-center, prospective cohort study
Source: BMC Med. 2020 Dec 14;18:390. doi: 10.1186/s12916-020-01875-5 (PMC7733739; doi:10.1186/s12916-020-01875-5)
Supplement: Supplementary file 1 — Additional file 1: Table S1. Characteristics of the patients who fulfil the sepsis-3 criteria for septic shock in the development set and validation sets. [file 12916_2020_1875_MOESM1_ESM.docx]

Table S1. Characteristics of the patients who fulfil the sepsis-3 criteria for septic shock in the development set and validation sets.

| Characteristics | Total set  (N = 516) | Development set (n = 248) | Validation set  (n = 268) | *p* value |
| --- | --- | --- | --- | --- |
| Age, years | 66.0 (60.0-75.0) | 67.0 (60.0-74.8) | 66.0 (59.0-75.0) | 0.75 |
| Male | 325 (62.5) | 157 (63.3) | 168 (62.7) | 0.88 |
| Hypertension | 178 (34.5) | 89 (35.9) | 89 (33.2) | 0.52 |
| Diabetes mellitus | 126 (24.4) | 70 (28.2) | 56 (20.9) | 0.05 |
| Cancer type |  |  |  | 0.66 |
| Gastrointestinal | 75 (14.5) | 29 (11.7) | 46 (17.2) |  |
| Hepatobiliary-pancreas | 182 (35.3) | 89 (35.9) | 93 (34.7) |  |
| Lung | 83 (16.1) | 41 (16.5) | 42 (15.7) |  |
| Gynecologic | 45 (8.7) | 23 (9.3) | 22 (8.2) |  |
| Urologic | 37 (7.2) | 19 (7.7) | 18 (6.7) |  |
| Others | 94 (18.2) | 47 (19.0) | 47 (17.5) |  |
| Focus of infection |  |  |  | 0.32 |
| Pneumonia | 116 (22.5) | 57 (23.0) | 59 (22.0) |  |
| Urinary tract infection | 69 (13.4) | 39 (15.7) | 30 (11.2) |  |
| Colitis | 71 (13.8) | 28 (11.3) | 43 (16.0) |  |
| Cholangitis/Cholecystitis | 145 (28.1) | 68 (27.4) | 77 (28.7) |  |
| Others/Unknown | 65 (12.6) | 35 (14.1) | 30 (11.2) |  |
| Multiple focus | 50 (9.7) | 21 (8.5) | 29 (10.8) |  |
| Vital signs at ED admission |  |  |  |  |
| Systolic BP, mmHg | 88.0 (74.0-110.0) | 89.0 (76.0-110.0) | 88.0 (73.3-109.8) | 0.57 |
| Diastolic BP, mmHg | 55.0 (47.0-66.0) | 55.0 (46.0-67.0) | 55.0 (47.0-65.8) | 0.65 |
| Heart rate/min | 117 (100-134) | 118 (100-132) | 117 (100-137) | 0.76 |
| Respiratory rate/min | 20.0 (18.0-24.0) | 20.0 (19.3-24.0) | 20.0 (18.0-24.0) | 0.26 |
| Body temperature, °C | 37.6 (36.8-38.6) | 37.7 (36.9-38.7) | 37.5 (36.7-38.6) | 0.16 |
| Altered mentation | 73 (14.1) | 42 (16.9) | 31 (11.6) | 0.08 |
| Laboratory values |  |  |  |  |
| White blood cell, /µL | 8300 (2710-16420) | 8050 (2593-16200) | 8690 (2975-16538) | 0.79 |
| Hemoglobin, g/dL | 10.2 (8.5-11.7) | 10.2 (8.4-11.6) | 10.3 (8.8-12.0) | 0.18 |
| Platelet count, x1000/µL | 132.5 (68.0-219.8) | 131.0 (66.0-207.5) | 134.5 (70.3-234.5) | 0.32 |
| PT, INR | 1.33 (1.20-1.54) | 1.34 (1.23-1.53) | 1.32 (1.17-1.55) | 0.19 |
| Albumin, g/dL | 2.7 (2.3-3.1) | 2.7 (2.3-3.1) | 2.7 (2.3-3.2) | 0.37 |
| BUN, mg/dL | 27.0 (19.0-40.6) | 28.0 (19.0-39.0) | 26.9 (19.0-41.1) | 0.71 |
| Creatinine, mg/dL | 1.39 (0.97-2.17) | 1.38 (0.98-2.19) | 1.40 (0.95-2.11) | 0.86 |
| CRP, mg/dL | 13.4 (5.5-21.8) | 14.1 (5.9-22.4) | 12.4 (5.2-21.3) | 0.52 |
| Lactic acid, mmol/L | 4.4 (3.1-6.5) | 4.4 (2.9-6.6) | 4.3 (3.2-6.5) | 0.69 |
| Severity score |  |  |  |  |
| SOFA score | 9.0 (7.0-12.0) | 9.0 (7.0-12.0) | 9.0 (7.0-12.0) | 0.23 |
| APACHE II score | 22.0 (17.0-29.0) | 22.0 (17.0-27.0) | 23.0 (17.0-30.0) | 0.33 |
| Quick SOFA score | 1.0 (1.0-2.0) | 1.0 (1.0-2.0) | 1.0 (1.0-2.0) | 0.82 |
| NEWS | 8.0 (5.3-10.0) | 8.0 (6.0-10.0) | 8.0 (5.0-10.0) | 0.57 |
| MEWS | 5.0 (4.0-7.0) | 5.0 (4.0-7.0) | 5.0 (4.0-7.0) | 0.94 |
| ICU admission | 353 (68.4) | 168 (67.7) | 185 (69.0) | 0.75 |
| 28-day mortality | 163 (31.6) | 77 (31.0) | 86 (32.1) | 0.80 |
| Values are presented as median (interquartile range) or number (percentage), as appropriate. | | | | |
| Abbreviations: APACHE, Acute Physiology and Chronic Health Evaluation; BP, blood pressure; BUN, blood urea nitrogen; CRP, C-reactive protein; ED, emergency department; ICU, intensive care unit; INR, international normalized ratio; MEWS, modified early warning score; NEWS, national early warning score; PT, prothrombin time; SOFA, Sequential Organ Failure Assessment. | | | | |
