# Supplementary material for: Development and validation of the VitaL CLASS score to predict mortality in stage IV solid cancer patients with septic shock in the emergency department: a multi-center, prospective cohort study
Source: BMC Med. 2020 Dec 14;18:390. doi: 10.1186/s12916-020-01875-5 (PMC7733739; doi:10.1186/s12916-020-01875-5)
Supplement: Supplementary file 2 — Additional file 2: Table S2. The C-indices for testing of the VitaL CLASS score and other pre-existing scoring systems in the patients who fulfil the sepsis-3 criteria for septic shock. [file 12916_2020_1875_MOESM2_ESM.docx]

Table S2. The C-indices for testing of the VitaL CLASS score and other pre-existing scoring systems in the patients who fulfil the sepsis-3 criteria for septic shock

| Scoring system | Development set | | | Validation set | | |
| --- | --- | --- | --- | --- | --- | --- |
|  | AIC | C-index (95% CI) | *p* value | AIC | C-index (95% CI) | *p* value |
| VitaL CLASS score | 730.0 | 0.806 (0.761-0.851) | reference | 887.6 | 0.702 (0.649-0.755) | reference |
| SOFA score | 772.8 | 0.714 (0.655-0.774) | 0.006 | 900.4 | 0.674 (0.614-0.733) | 0.43 |
| APACHE II score | 774.5 | 0.702 (0.642-0.761) | 0.002 | 897.2 | 0.674 (0.615-0.733) | 0.46 |
| Quick SOFA score | 809.4 | 0.615 (0.551-0.678) | <0.001 | 927.9 | 0.569 (0.512-0.626) | <0.001 |
| NEWS | 802.5 | 0.644 (0.580-0.709) | <0.001 | 928.7 | 0.562 (0.501-0.623) | <0.001 |
| MEWS | 820.0 | 0.536 (0.468-0.604) | <0.001 | 930.6 | 0.534 (0.472-0.597) | <0.001 |

Abbreviations: AIC, Akaike information criterion; APACHE, acute physiology and chronic health evaluation; CI, confidence interval; MEWS, modified early warning score; NEWS, national early warning score; SOFA, sequential organ failure assessment; VitaL CLASS, vital signs-lung cancer-lactate-albumin in septic shock.
